# The Complicated Case of Staphylococcus aureus Bacteremia Associated With Delayed-Onset Prosthetic Joint Infection: A Case Report and Review of Management Strategies

**DOI:** 10.7759/cureus.66854

**Published:** 2024-08-14

**Authors:** Shaniah S Holder, Ehizele P Itama, Samuel N Ikediashi, Abigail Greaves, Alaerebo S Malvan-Iyalla, Frank Hsu

**Affiliations:** 1 Medicine, American University of Barbados School of Medicine, Bridgetown, BRB; 2 Internal Medicine, Insight Hospital and Medical Center, Chicago, USA

**Keywords:** prosthetic joint infection (pji), staphylococcus aureus bacteremia, infective endocarditis, methicillin-sensitive staphylococcus aureus, staphylococcus aureus

## Abstract

*Staphylococcus aureus* (*S. aureus*), an opportunistic Gram-positive bacterium, is notorious for causing a plethora of clinical diseases. While it does not typically infect healthy skin, *S. aureus* infections are prevalent in both community-acquired and hospital-acquired settings. Rheumatoid arthritis (RA), a chronic autoimmune disease characterized by joint inflammation and progressive bone erosion, can be managed medically and, in moderate to severe cases, surgically through arthroplasty. Complications of arthroplasty include wound infection, blood clots, stiffness, and infection around the prosthesis. Prosthetic joint infections (PJIs) are a rare complication of arthroplasty, commonly caused by aerobic Gram-positive bacteria. These infections can lead to bacteremia, precipitating a cascade of adverse clinical sequelae. This report aims to explore the etiology of delayed-onset PJIs, the underlying pathophysiology of this condition leading to bacteremia, the complications of *S. aureus* bacteremia, and the management strategies employed to treat PJIs and complicated cases of *S. aureus* bacteremia resulting from PJIs.

## Introduction

*Staphylococcus aureus* (*S. aureus*) is a Gram-positive bacterium that is responsible for a vast range of diseases. It can cause both community-acquired and hospital-acquired infections [1]. This bacterium is commonly found in the environment and constitutes part of the normal human flora residing on the skin and mucosal membranes [1]. A study conducted using the Department of Defense TRICARE network found that skin infections accounted for a higher incidence rate of *S. aureus* infections as compared to bacteremia [2]. Additionally, *S. aureus *is implicated with other infections such as infective endocarditis (IE), osteomyelitis, and septic arthritis [2].

Rheumatoid arthritis (RA), a systemic autoimmune disease characterized by inflammatory arthritis and extra-articular involvement, is more prevalent in the United States and Europe [3]. Epidemiological data reveal that women are two to three times more likely to present with RA than men and the risk increases with age [3]. The hallmark features of RA include gradually worsening joint pain, swelling, and morning stiffness over weeks to months. The recommended therapy for RA includes non-steroidal anti-inflammatory drugs (NSAIDs), steroids, disease-modifying antirheumatic drugs (DMARDs), and biologics like infliximab [3]. However, in refractory cases and in patients with advanced disease progression in the hip joint, total hip arthroplasty has been proven to be successful in reducing pain and improving joint function and mobility [4]. 

Prosthetic joint infection (PJI), also known as periprosthetic infection, is defined as an infection of the joint prosthesis and surrounding tissue [5]. Knee joint PJIs are more common than hip joint PJIs with an incidence rate of 2% and 1%, respectively [6]. PJI is classified into early-onset (less than three months postoperative), delayed-onset (more than three months but less than one year postoperative), and late-onset (more than one year postoperative) [5,6]. Risk factors for developing PJI include prior surgery at the arthroplasty site, current bacteremia or sepsis, and a previous or active infection at the current surgical site [6]. Delayed-onset PJI is usually caused by coagulase-negative *Staphylococcus*, *Cutibacterium acnes,* and enterococci, whereas *S. aureus *is a less frequent cause [6]. PJIs can present with an array of symptoms including joint pain and signs of acute infection such as fever, skin erythema, and edema. Delayed-onset PJIs can present with chronic joint pain, sinus tract formation, and possible loosening of the hardware [7].

We present an interesting case of a 64-year-old female with a history of severe RA who presented with a complicated case of *S. aureus* bacteremia due to a delayed-onset PJI 10 months after arthroplasty. 

## Case presentation

The patient, a 64-year-old female, presented to the emergency department (ED) with dysuria, increased urinary frequency, foul-smelling dark urine, and altered mental status (AMS) over the past four days. Per family members, her baseline mentation was alert and oriented to person, place, and time (AAO ×3). However, she subsequently became agitated and was only oriented to person (AAO ×1). 

The patient's history is significant for end-stage osteoarthritis secondary to severe RA being managed with intravenous (IV) infliximab bimonthly with the most recent one being one month ago. Notably, she underwent a right hip total arthroplasty 10 months prior to presentation. However, she did not attend any follow-up appointments or obtain the recommended post-procedure imaging. During the physical assessment, the patient did not exhibit signs of acute distress but expressed pain upon palpation in the suprapubic and lower back areas. Her vital signs revealed systolic hypertension with a blood pressure reading of 180/65 mmHg (normal value: ~120/80 mmHg) and tachycardia with a heart rate of 110 beats per minute (reference range: 60-100 beats per minute).

Blood labs were significant for mild normocytic anemia, severe leukocytosis, elevated procalcitonin, and hyperlactatemia. Table 1 highlights her significant lab findings.

**Table 1:** Patient's significant lab findings on presentation to the emergency department Hb: hemoglobin; MCV: mean corpuscular volume; WBC: white blood cell

Laboratory parameter	Value	Reference range
Hb	11 g/dL	11.6-15.1 g/dL
MCV	88 fL	80-100 fL
WBC count	20,000 cells/µL	4,000-11,000 cells/µL
Procalcitonin	5.03 ng/mL	<0.1 ng/mL
Lactate	2.8 mmol/L	<2 mmol/L

Urinalysis results returned with positive nitrites and leukocyte esterase, moderate bacteriuria, and a white blood cell count of 26-50 cells indicating a urinary tract infection (UTI). Due to AMS, meeting two out of four criteria for systemic inflammatory response syndrome (SIRS) and the presence of a source of infection, the patient was diagnosed with acute metabolic encephalopathy secondary to sepsis. Initial treatment in the ED included aztreonam and vancomycin. Upon admission to the ICU for further monitoring, the patient was placed on vancomycin, metronidazole, and cefepime. 

**Figure 1:**
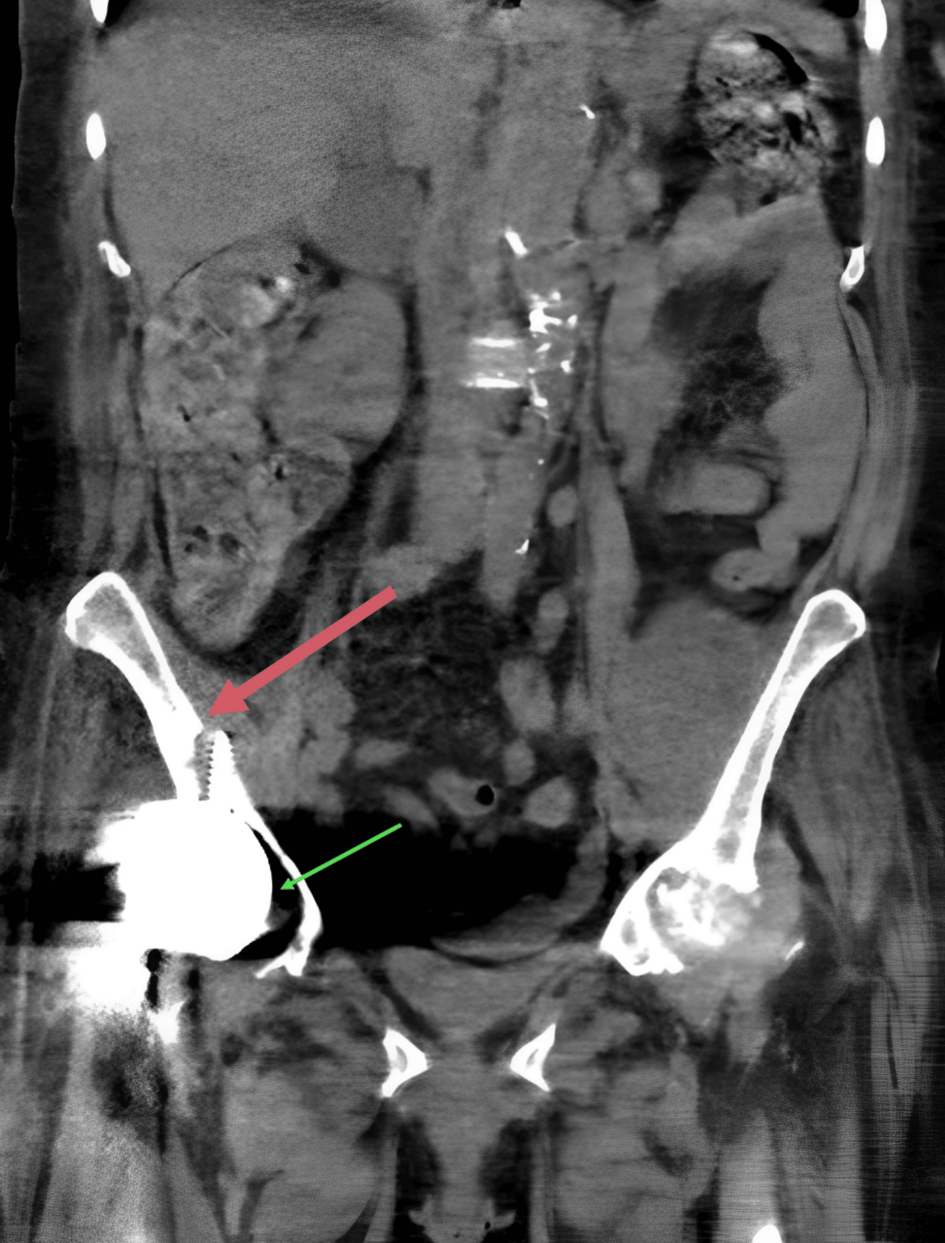
Perihardware lucency surrounding the superior screw of the right hip arthroplasty with the tip extending beyond the medial cortical border (red thick arrow). Also, notable superolateral subluxation of the acetabular cup (green thin arrow)

A computed tomography scan of the abdomen and pelvis ruled out pyelonephritis. However, unexpected findings included perihardware lucency surrounding the superior screw of the right hip arthroplasty with the tip of the screw extending beyond the medial cortical border which is associated with loosening and/or infection. Figure 1 shows these findings.

Cultures of the urine, hip joint aspirate, and blood were taken. Urine culture demonstrated growth of not only *Klebsiella pneumoniae* but also >100,000 colony-forming units per milliliter (cfu/ml) of *S. aureus*. The hip joint aspirate was purulent, and two cultures revealed the presence of *S. aureus*. Blood cultures from two IV sites were similarly remarkable, revealing the presence of *S. aureus* indicating *S. aureus *bacteremia. Antibiotic sensitivity testing results revealed methicillin-sensitive *S. aureus *(MSSA) with sensitivity to many antibiotics. Table 2 shows the results of the antibiotic sensitivity test. 

**Table 2:** Results of the Staphylococcus aureus antibiotic sensitivity test from the blood specimen S: sensitive

Antibiotic	Result
Amoxicillin/clavulanate	S
Ampicillin/sulbactam	S
Clindamycin	S
Daptomycin	S
Erythromycin	S
Gentamicin	S
Oxacillin	S
Tetracycline	S
Trimethoprim/sulfamethoxazole	S
Vancomycin	S

**Figure 2:**
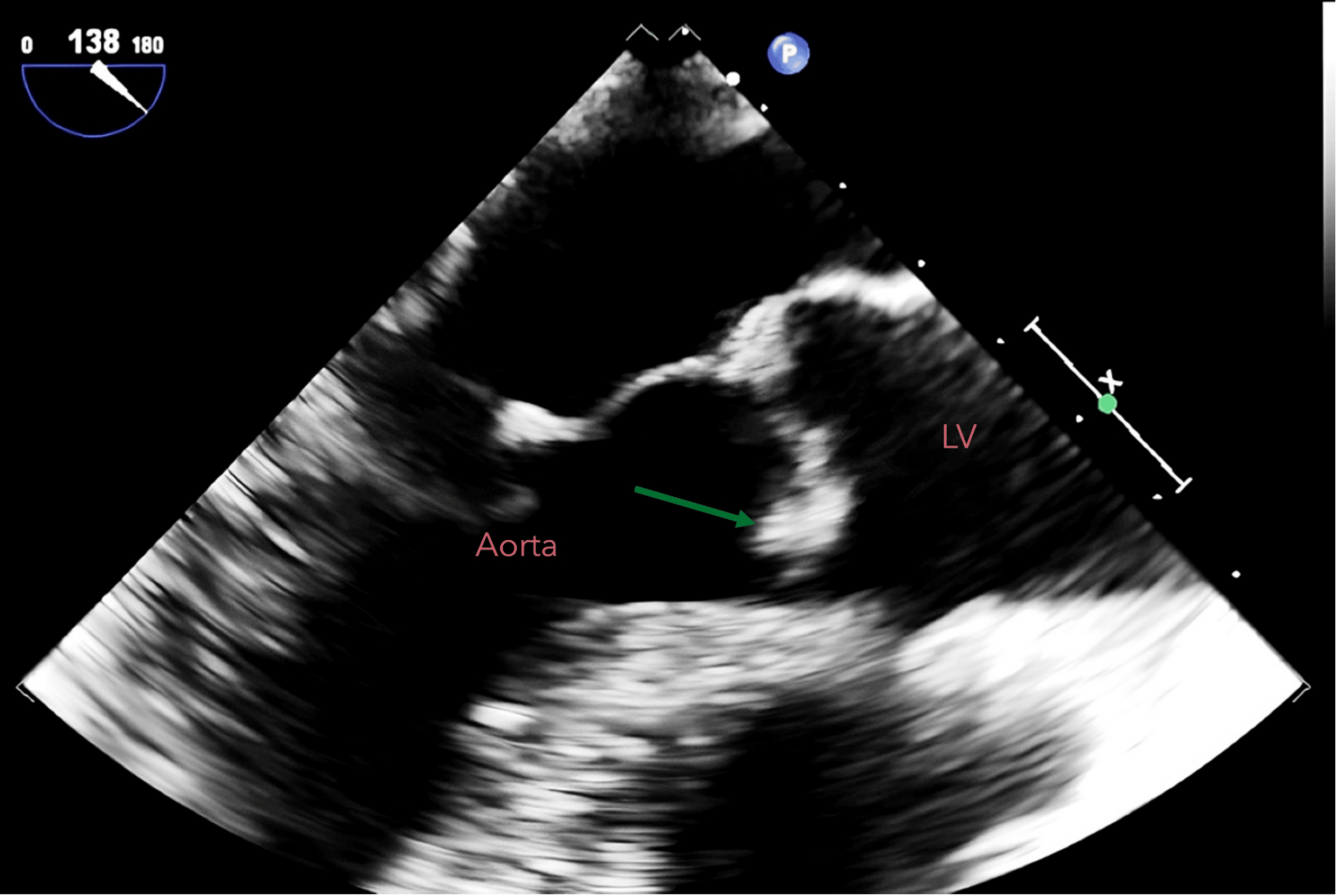
Echocardiography showing a 2-mm vegetation on the right coronary cusp of the aortic valve (green arrow)

In the context of *S. aureus *bacteremia, a transesophageal echocardiography (TEE) was performed to assess the heart valves for potential IE. The findings revealed a 2-mm vegetation on the right coronary cusp of the aortic valve, accompanied by moderate aortic regurgitation. Figure 2 depicts the vegetation on the valve, while Video 1 highlights the color flow Doppler demonstrating blood regurgitation from the aorta to the left ventricle.

**Video 1:** Color flow Doppler echocardiogram showing aortic regurgitation (blue color indicating regurgitant flow directed away from the transducer)

Given the patient's history of arthroplasty more than three months but less than one year prior to presentation and imaging showing loosening of the hardware, it was suspected that the patient had a delayed PJI which led to *S. aureus* bacteremia and subsequent IE. Orthopedic surgery was consulted and concurred with this suspicion. Based on Duke's criteria, the patient met the two major criteria confirming the definitive diagnosis of IE. Table 3 highlights modified Duke's criteria.

**Table 3:** Modified Duke's criteria for the diagnosis of infective endocarditis

Criteria	Description
Major clinical criteria	Blood cultures positive for endocarditis
	Evidence of endocardial involvement
Minor clinical criteria	Predisposing heart condition or injection drug use
	Fever
	Vascular phenomena
	Immunologic phenomena
	Microbiological evidence
Diagnostic criteria	Definite diagnosis: Two major criteria or one major and three minor criteria or five minor criteria
	Possible diagnosis: One major and one minor criteria or three minor criteria

Metronidazole and cefepime were replaced with meropenem, while vancomycin was continued. 

Over the next four weeks, the patient's clinical status improved with the antibiotic regimen, and repeat blood and urine cultures showed no bacterial growth. Once medically stable, the infected hardware components were removed, and an antibiotic spacer was placed. This approach allowed for infection control while maintaining joint stability until her future hip revision. Subsequently, she was discharged to a nursing facility and underwent hip revision with prosthetic joint placement months later. 

## Discussion

PJI is a serious complication of prosthetic implantation. The underlying etiology of PJIs includes intraoperative contamination in early- and delayed-onset PJI and hematogenous bacterial dissemination to the joint from other parts of the body in late-onset PJI [7]. Biofilm development plays a critical role in the pathophysiology of PJIs. Chronic, low-level populations of adherent bacteria often exhibit slow growth and evade host defenses through intracellular persistence and/or the production of extracellular polysaccharides [8]. These polysaccharides form a biofilm, shielding the bacteria and contributing to tissue damage [7]. Biofilm formation attaches to surfaces such as medical devices or tissue via proteins or teichoic acids, followed by multiplication and secretion of an extracellular matrix, promoting adherence and infection [8].

The 2018 International Consensus Meeting (ICM) diagnostic criteria is one of the widely adopted criteria within the orthopedic community as a standard for establishing a PJI diagnosis. PJI is diagnosed when one major or four out of six minor criteria are met. Table 4 highlights the criteria factors [9]. 

**Table 4:** The International Consensus Meeting diagnostic criteria for establishing a diagnosis of PJI PJI: prosthetic joint infection; CRP: C-reactive protein; ESR: erythrocyte sedimentation rate; WBC: white blood cell; PMN: polymorphonuclear

Criteria	Factors
Major criteria	Two positive periprosthetic cultures with phenotypically identical organisms
	A sinus tract communicating with the joint
Minor criteria	Elevated CRP and ESR
	Elevated synovial fluid WBC count or ++ change on leukocyte esterase test strip
	Elevated synovial fluid PMN cells
	Presence of purulence in the affected joint
	Positive histologic analysis of periprosthetic tissue
	A single positive culture

Radiography offers limited sensitivity and specificity for PJI; findings include joint effusion or malalignment, bone-cement-metal interface lucencies, periosteal reactions, periprosthetic bone resorption, transcortical sinus tracts, or patchy osteolysis [10]. In this case, imaging revealed perihardware lucency and hardware loosening. Periprosthetic cultures were positive for *S. aureus* meeting one major criterion, thus confirming her PJI. 

Due to the possible complications associated with prosthetic joint implantation, postoperative follow-ups are important for adequate monitoring for early warning signs of complications [11]. In the case of our patient, there was a lack of proper follow-up which led to the development of delayed-onset PJI. The incidence of delayed-onset PJI caused by *S. aureus* varies, and bacteremia involving this organism can result in significant clinical complications. This organism has the potential to spread to other organs leading to embolic stroke, brain abscess, pneumonia, IE, and vertebral osteomyelitis [12]. Prosthetic devices in persons with joint inflammation act as a blood pipeline allowing bacteria into the intravascular space via various mechanisms. Bacterial enzymes and virulence factors help *S. aureus* invade surrounding tissues and penetrate blood vessels while evading the host's immune defenses [12]. The production of an antiphagocytic capsule, isolation of host antibodies or antigen masking by protein A, biofilm formation, intracellular survival, and blocking leukocyte chemotaxis are mechanisms responsible for the circumvention of the host body's immune system [13]. Inflammation in the joint leads to increased vascular permeability allowing bacteria to pass through vessel walls and biofilm disruption can facilitate the movement of bacteria into the surrounding tissues and subsequently the bloodstream [12]. 

There are many risk factors for the development of bacteremia from a PJI. Notably, one of the main factors is immunosuppression [14]. Infliximab which was used in this patient is an immunosuppressant used in the treatment of RA. Its immunosuppressive effects increase susceptibility to infections which could have also played a part in our patient's complicated bacteremia from delayed-onset PJI. Other factors include poor nutritional status, obesity, smoking, diabetes mellitus, advanced age, and other factors that can impair wound healing [14].

*S. aureus *bacteremia can lead to a series of severe complications. In this case, the patient developed IE. The overall incidence of IE is increasing, with two-thirds of all cases attributed to *S. aureus* [15]. In IE, *S. aureus* binds to extracellular matrix proteins and fibronectin via bacterial cell wall-associated proteins such as fibrinogen-binding proteins, clumping factors, and teichoic acids [13]. The diagnostic approach for IE involves a combination of clinical features, laboratory tests, and imaging studies. Echocardiography is the optimal tool in the initial work-up of suspected IE. TEE has a higher diagnostic accuracy than transthoracic echocardiography and is superior for detecting small vegetations (<5 mm) [15]. *S. aureus* IE predominantly affects the aortic valve, with approximately 20% of cases involving the tricuspid valve. However, in IV drug users, the incidence of tricuspid valve IE is higher, ranging from 30% to 70% of cases [15]. Duke's criteria provide clinical guidelines for diagnosing IE, with an average sensitivity of 80% [16]. A definite diagnosis requires either both major criteria, one major criterion, and three minor criteria or five minor criteria [16]. In this patient's case, meeting both major criteria solidified the diagnosis of IE.

Treatment of localized PJIs includes microorganism-specific IV antibiotic therapy for at least six weeks and surgical replacement of the infected hardware to eliminate the infection, restore joint function, and relieve symptoms [6]. Antibiotics effective against bacterial clusters (biofilms), like rifampin and fluoroquinolones, are commonly used as first-line treatments [14]. Other potential oral medications include minocycline, linezolid, and trimethoprim-sulfamethoxazole [14]. The preferred surgical treatment in the United States and the method used in this patient is the two-step implant exchange method, where the infected joint is removed and a temporary antibiotic-filled spacer is placed for two to eight weeks, followed by joint replacement [17]. Before the final replacement, patients must show improvement, normal blood test results, and no signs of infection two weeks after completing antibiotic treatment [17].

Treatment of *S. aureus* bacteremia depends on the bacteria's resistance profile. Beta-lactam antibiotics like oxacillin and flucloxacillin are prescribed for milder MSSA infections, while severe methicillin-resistant *S. aureus* (MRSA) infections often require vancomycin [15]. In this patient, vancomycin was administered with meropenem due to the case's complexity. A study by Tong et al. in 2015 reported an 80% case fatality rate for untreated *S. aureus* infections, decreasing to 15-50% with antibiotics [18]. The exact figure varies based on age, overall health, and strain-specific resistance.

The complex nature of *S. aureus* bacteremia from a PJI justifies the need for rapid, accurate diagnostic testing combined with an interdisciplinary approach. Close post-arthroplasty surveillance and patient education about the symptoms of infection have critical importance in ensuring prompt recognition, diagnosis, intervention, and prevention of complications.

## Conclusions

Delayed-onset PJIs are rarely caused by *S. aureus*. However, *S. aureus* is a formidable pathogen that, when involved, can lead to severe consequences such as bacteremia. The risk of complicated *S. aureus* bacteremia is significantly elevated in elderly and immunocompromised individuals, as seen in this case. This bacteremia can further complicate matters by leading to conditions such as IE, a serious infection of the heart valves with dire consequences. This chain of adverse effects stemming from a joint infection highlights the importance of early detection and intervention to mitigate these risks. 

Patient adherence to follow-up care after arthroplasty is crucial in reducing the incidence of PJIs and their detrimental complications. Regular imaging and prompt management of any signs of infection can decrease the risk of developing *S. aureus* bacteremia and subsequent complications like IE. Implementing strict postoperative protocols and educating patients on recognizing early symptoms of infection are essential strategies to prevent these potentially life-threatening complications and improve quality of life. 
